# My NGS and Other Animals

**DOI:** 10.3390/ijms26094111

**Published:** 2025-04-26

**Authors:** Marsel Kabilov

**Affiliations:** Institute of Chemical Biology and Fundamental Medicine, Siberian Branch of the Russian Academy of Sciences, 630090 Novosibirsk, Russia; kabilov@niboch.nsc.ru

High-throughput sequencing is one of a number of omics technologies that generates huge amounts of data in the form of nucleotide sequences. Currently, it is primarily associated with NGS, but, in reality, the era of high-throughput sequencing began in the 90s with the upgrade of the Sanger method, which uses fluorescent dyes and capillary electrophoresis for product analysis, and with the introduction of short-gun technology for obtaining fragment libraries. The work of huge teams of researchers and the use of factories of 96-capillary genetic analyzer sequencing according to Sanger resulted in the successive appearance of the first genomes: the bacterium *Haemophilus influenzae* [1] in 1995, the yeast *Saccharomyces cerevisiae* [2] in 1996, the nematode *Caenorhabditis elegans* [3] in 1998, and eventually the human genome in 2001 [4,5]. All these genomic projects, the human genome project being the first and foremost, became the basis for the emergence of high-throughput sequencing platforms that produce short-reads (Illumina, MGI, etc.) and long-reads (PacBio, Nanopore), collectively referred to as Next Generation Sequencing.

The emergence of NGS platforms has dramatically reduced the cost of genomic sequencing. The cost of the first bacterial genome, sequenced on the first NGS platform 454, was orders of magnitude cheaper than Sanger sequencing [6]. Contrary to Moore’s law, the cost of NGS has been falling at an accelerated pace and new forecasts promise a human genome for $100 [7]. However, it is necessary to distinguish between de novo genome assembly, carried out for most of the first genomes, and resequencing, which comes down to mapping reads to a previously assembled genome. Obtaining de novo genomes of viruses and bacteria has already become a completely common approach. I believe the same fate awaits the assembly of eukaryotic genomes to chromosomes in the near future.

High-throughput sequencing as a tool for studying biological systems is, in some senses, becoming an increasingly routine tool for molecular biologists and geneticists. Primarily, this is due to the fact that genome and transcriptome sequencing, with some reservations, has become maximally simplified. Further development of NGS is becoming largely associated with methods that will allow researchers to address more specific questions.

This Special Issue of *IJMS* “Selected Papers from the HSG-2022 Conference” includes 10 research papers that present only a part of the huge variety of NGS approaches. Most studies had genome structure as their focus. The development of NGS methods, in particular ONT (Oxford Nanopore Technologies), which allows the obtainment of long reads, has made obtaining circular bacterial genomes a fairly routine method. The article by Khrenova et al. describes the de novo assembly of the *E. coli* genome, carried out using ONT, and the results of studying the possibility of detecting SNPs and gene deletions causing antibiotic resistance (contribution 1). The impact of such parameters as reads quality, coverage, and type of assembler on assembly and mutation detection was analyzed. Another article in the Special Issue is devoted to the use of ONT for de novo assembly and analysis of 50 Russian strains of *Neisseria gonorrhoeae* (contribution 2). Comparison of the obtained genomes with those already known from databases allowed researchers to identify mobile elements associated with genome rearrangements. The third article by Gladkov et al. explores the use of ONT for comparing communities of microorganisms with lignocellulose degradation properties (contribution 3). Metagenomic analysis of four natural consortia revealed major bacterial participants, as well as degradation enzymes, which were similar in all microbiome variants.

In the section of works related to the use of NGS for cytogenetic purposes, two articles were presented. The first article describes the low-coverage sequencing of the Nile Crocodile genome, performed for the first time (contribution 4) not for de novo assembly, but for the identification of tandem repetitive DNAs and LTR retrotransposons. The study found that repeats were localized on chromosomes using fluorescence in situ hybridization, which made it possible to clarify the localization and composition of pericentromeric regions. In the second work, Tishakova et al. (contribution 5) studied the veiled chameleon genome. Using a cell sorter, individual chromosomes were obtained, and DNA libraries for NGS were prepared. Mapping reads from chromosome-specific libraries to three already known reptile genomes made it possible to identify the putative sex chromosome pair.

The last section of this Special Issue includes articles related to the use of transcriptome analysis. Two articles present purely bioinformatic works performed on data available from the NGS databases. Thus, in the work of Dvorianinova et al. (contribution 6), genes involved in fatty acid synthesis in flax were identified, for which both genomic data—used to assess the level of polymorphisms in the sought-after genes—and transcriptome data—to assess their expression in different tissues—were used. The second bioinformatics work describes the comparison of gene expression levels in three types of liver cell lines (HepG2, Huh7, and Hep3B) (contribution 7). Significant differences in metabolic pathways associated with oxidative phosphorylation, cholesterol metabolism, and DNA damage were revealed.

In the experimental work of Zhuravlev et al., the observed changes in mRNA and snoRNA expression were analyzed in the A549 human cell line infected with influenza virus A/Puerto Rico/8/1934 (H1N1) (contribution 8). It was demonstrated that the pattern of snoRNA expression in cells changes significantly during virus infection. Kossinova et al. (contribution 9) reports the results of the study using several methods of transcriptome analysis for the HEK293T cell line. Firstly, the PAR-Clip technique was used to identify about 100 GC-reached mRNAs that bind the RNA cytosine C5 methyltransferase NSUN2. Secondly, using a combination of standard RNA-seq and less common Poly-Ribo-Seq, which allows the authors to assess the change in expression of only translated mRNAs, differentially expressed genes were investigated in the case of NSUN2 expression suppression using siRNA. Komarova et al. (contribution 10) studied the efficiency of fluorescent protein translation in *E. coli*, depending on the 5′-UTR sequence. The plasmid library contained 648 natural 5′-UTRs, the efficiency of which was ranked using a cell sorter depending on the fluorescence level (Flow-seq). The obtained results were in good agreement with Ribo-seq data obtained in other studies.

In conclusion, all articles included in this Special Issue of *IJMS* demonstrate the potential of high-throughput sequencing methods to address a wide range of very different biological problems. As the Guest Editor, I would like to thank all the authors who submitted their manuscripts to the Special Issue after presenting their works at HSG-2022.

## References

[1] Fleischmann R.D., Adams M.D., White O., Clayton R.A., Kirkness E.F., Kerlavage A.R., Bult C.J., Tomb J.-F., Dougherty B.A., Merrick J.M. (1995). Whole-Genome Random Sequencing and Assembly of *Haemophilus influenzae* Rd. Science.

[2] Goffeau A., Barrell B.G., Bussey H., Davis R.W., Dujon B., Feldmann H., Galibert F., Hoheisel J.D., Jacq C., Johnston M. (1996). Life with 6000 Genes. Science.

[3] The C. elegans Sequencing Consortium (1998). Genome Sequence of the Nematode *C. Elegans*: A Platform for Investigating Biology. Science.

[4] Venter J.C., Adams M.D., Myers E.W., Li P.W., Mural R.J., Sutton G.G., Smith H.O., Yandell M., Evans C.A., Holt R.A. (2001). The Sequence of the Human Genome. Science.

[5] Lander E.S., Linton L.M., Birren B., Nusbaum C., Zody M.C., Baldwin J., Devon K., Dewar K., International Human Genome Sequencing Consortium, Whitehead Institute for Biomedical Research, Center for Genome Research (2001). Initial Sequencing and Analysis of the Human Genome. Nature.

[6] Margulies M., Egholm M., Altman W.E., Attiya S., Bader J.S., Bemben L.A., Berka J., Braverman M.S., Chen Y.-J., Chen Z. (2005). Genome Sequencing in Microfabricated High-Density Picolitre Reactors. Nature.

[7] Satam H., Joshi K., Mangrolia U., Waghoo S., Zaidi G., Rawool S., Thakare R.P., Banday S., Mishra A.K., Das G. (2023). Next-Generation Sequencing Technology: Current Trends and Advancements. Biology.

